# Multiplex enzyme activity imaging by MALDI-IMS of substrate library conversions

**DOI:** 10.1038/s41598-020-72436-2

**Published:** 2020-09-23

**Authors:** Oliver Klein, Akvile Haeckel, Ulf Reimer, Grit Nebrich, Eyk Schellenberger

**Affiliations:** 1 Department of Radiology, Charité – Universitätsmedizin Berlin, Charitéplatz 1, 10117 Berlin, Germany; 2 Berlin-Brandenburg Center for Regenerative Therapies, Charité – Universitätsmedizin Berlin, Augustenburger Platz 1, 13353 Berlin, Germany; 3 JPT Peptide Technologies GmbH, Volmerstraße 5, 12489 Berlin, Germany

**Keywords:** Biochemistry, Biological techniques, Cancer, Molecular biology, Systems biology, Biomarkers, Medical research, Molecular medicine

## Abstract

Enzymes are fundamental to biological processes and involved in most pathologies. Here we demonstrate the concept of simultaneously mapping multiple enzyme activities (EA) by applying enzyme substrate libraries to tissue sections and analyzing their conversion by matrix-assisted laser desorption/ionization (MALDI) imaging mass spectrometry (IMS). To that end, we spray-applied a solution of 20 naturally derived peptides that are known substrates for proteases, kinases, and phosphatases to zinc-fixed paraffin tissue sections of mouse kidneys. After enzyme conversion for 5 to 120 min at 37 °C and matrix application, the tissue sections were imaged by MALDI-IMS. We could image incubation time-dependently 16 of the applied substrates with differing signal intensities and 12 masses of expected products. Utilizing inherent enzyme amplification, EA-IMS can become a powerful tool to locally study multiple, potentially even lowly expressed, enzyme activities, networks, and their pharmaceutical modulation. Differences in the substrate detectability highlight the need for future optimizations.

## Introduction

Enzymes are biological catalysts that are key-players in the metabolism and signal transduction of cells and organisms. Hence, their balance and regulation are essential for health, whereas alterations are involved in most diseases including cancer, neurodegenerative diseases, and cardiovascular disorders. As nano-engines of the body, enzymes are known to catalyze thousands of biochemical reactions^1^, making them an important field of biomedical research.

The ability to measure enzyme activities in vivo or ex vivo or, more specifically, to spatially map enzyme activities in tissue slices is of highest interest^2–4^. Based on fluorescence quenching of dyes with different fluorescence spectra, optical probes have the potential to report activities of several proteases but, due to spectral overlap, are limited to a few channels that can be discriminated by fluorescence microscopes^2,5,6^. Thus, there is an unmet need for technological improvements and developments to enable large-scale, simultaneous analysis of multiple enzymes in tissue specimens including other enzyme classes such as kinases and phosphatases.

Matrix-assisted laser desorption/ionization (MALDI) imaging mass spectrometry (IMS) has become a powerful tool to explore the molecular compositions of biological tissue sections. It allows label-free rapid localization of biomolecules (metabolites, drugs, lipids, peptides, and proteins) directly in tissue specimens without prior knowledge of their presence^7^. IMS has matured to a degree that now enables personalized pathology based on protein, peptide^8,9^, lipid^10,11^, and glycan^12,13^ signatures as well as drug monitoring directly from tissue specimens^14^.

**Figure 1:**
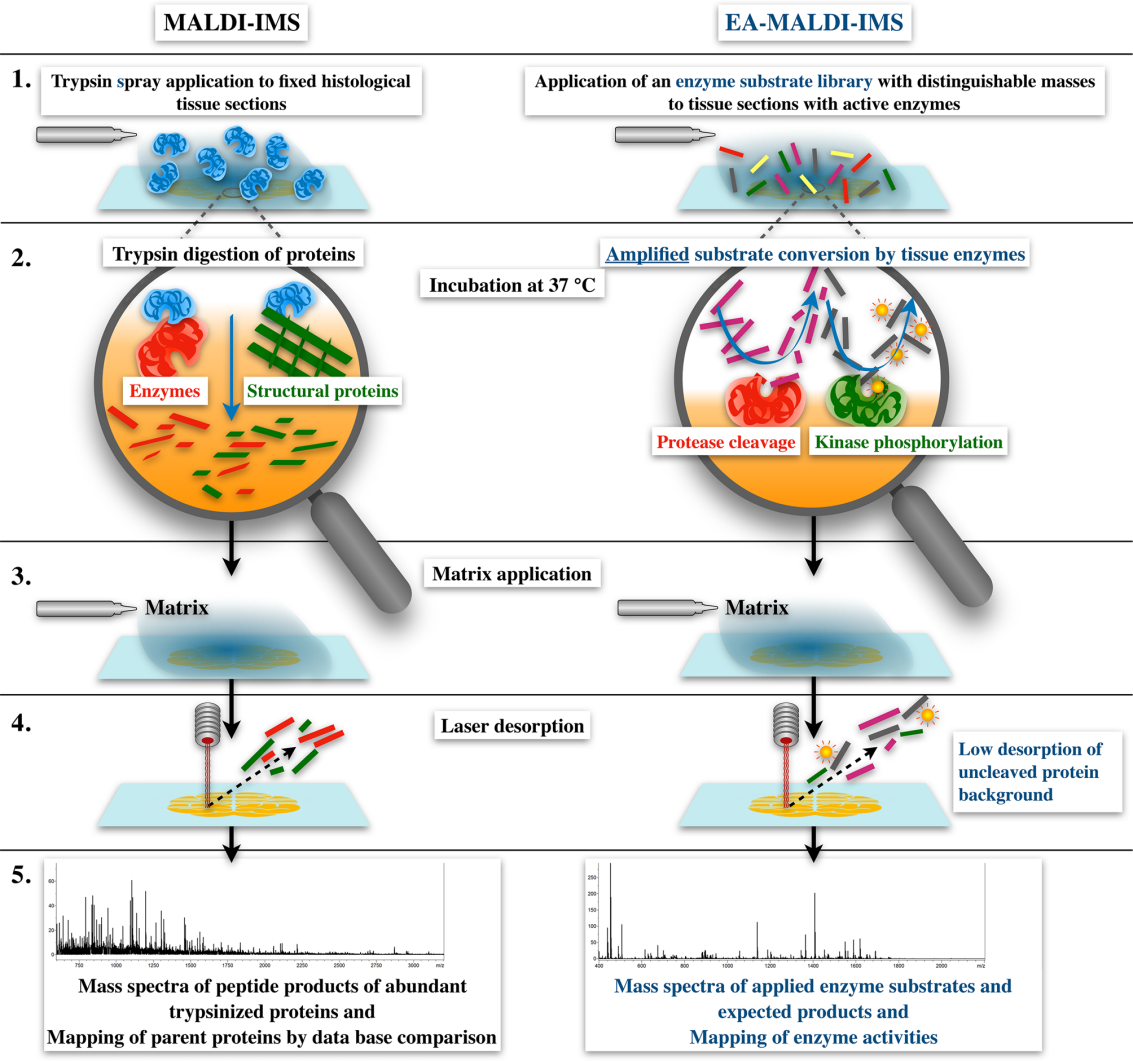
Comparison of typical trypsin-based MALDI-IMS and enzyme activity (EA)-MALDI-IMS. In conventional MALDI-IMS, trypsin is applied to fixed tissue sections in step 1 to digest proteins into well-desorbable, defined peptides in step 2. In contrast, EA-MALDI-IMS, or EA-IMS for short, is performed by applying a library of known substrates to tissue sections with active enzymes in step 1, which are then enzymatically converted by the enzymes present in the tissues during the incubation in step 2. Steps 3 to 5 are the same with both techniques except that, in EA-IMS, the resulting mass spectra are analyzed for the expected masses of substrates and their enzyme conversion products. The resulting maps can be substrate, product, or mathematically converted substrate/product maps.

For in-depth elucidation of interrelated enzyme activities and signaling pathways, it is highly desirable to have a tool that enables us to simultaneously image multiple enzyme activities in single sections of fixed tissue. For this purpose, we have reversed the MALDI-IMS procedure and replaced the solution that is usually spray-applied for on-tissue protein/glycan digestion (trypsin, PNGase) by a solution containing a library of naturally derived peptides that are known enzyme substrates (Fig. 1). Previous studies show that MALDI imaging is a valuable tool for investigating spatial enzyme activities. However, these studies were limited to the exploration of single natural peptide substrates and frozen tissues^15–17^ or phospholipids^18^. Here we explore the potential of MALDI-IMS for mapping multiple enzyme activities in zinc-fixated, paraffin-embedded tissues by spray applying enzyme substrate libraries of naturally derived, but artificial peptides.

These synthetic peptides (or potentially other substrates) have several advantages: (a) their concentration depends only on the applied substrates without natural tissue background; (b) they can be chosen/designed with lengths and masses that have low tissue background signal; (c) their sequence and structure can be optimized for synthesis of peptides with optimal MALDI-IMS properties, e.g., for desorption and ionization; (d) even synthesis of substrates that include non-amino acid chemical moieties for best MALDI properties is possible. Taken together, artificial peptides can be optimized to serve different purposes, e.g., to reliably image a maximum number of enzyme activities or to image particular very lowly expressed enzyme activities.

After application of the library and during subsequent incubation at 37 °C, the enzymes present in the tissue section convert the substrates into expected products with their predicted masses. As for trypsin digestion, a hydrophobic matrix is added in the fourth step. Following this step, the applied enzyme substrates and their resulting enzyme conversion products are analyzed by MALDI-IMS. In order to preserve enzyme activities, the tissue samples need to be embedded and fixed in a biocompatible way, which can be done, e.g., by tissue freezing or by zinc or ethanol fixation of paraffin-embedded tissues for better preservation of morphology^2,6^. While, in the past, MALDI-IMS was used to analyze the conversion of single substrates ex vivo^15–18^ and in sections of 3D cell culture organoids^19^, here we propose the development of extensive substrate libraries for future MALDI-IMS imaging of complex enzyme networks.

To demonstrate the concept of enzyme activity (EA) MALDI-IMS we synthesized naturally derived peptides with different masses that are known substrates for proteases, kinases, and phosphatases. A mixture of these peptides was spray-applied as a mixture to zinc-fixed tissue sections of kidneys, incubated in a humidity chamber, and imaged with MALDI-IMS.

## Methods

### Chemicals

Unless mentioned otherwise, chemicals were purchased form Merck KGaA (Darmstadt, Germany).

### Tissue preparation and zinc fixation

To prevent protein denaturation and maintain enzyme activity, the tissue samples were zinc-fixed as described previously^2,6^.

All experiments on mice were performed in accordance with relevant guidelines and regulations and animal housing and handling was approved by the State Office of Health and Social Affairs (LAGeSo), Berlin, Germany (approval number G0176/17). Whole kidneys of deeply anesthetized and subsequently exsanguinated wild-type mice were perfused with sterile 0.9% sodium chloride solution and fixed in zinc fixative (BD Pharmingen, Heidelberg, Germany) at room temperature for about 30 h and then washed in Millipore water for 1 h. After dehydration, the tissues were embedded in paraffin using the following procedure: 2 h 70% ethanol; 2 h 80% ethanol; 2 × 2 h 96% ethanol; 3 × 2 h 100% ethanol; 2 h 90% xylene + 10% ethanol; 2 × 2 h 100% xylene; 2 × 2 h melted paraffin at 60 °C. Following these steps, the fixed tissue was embedded in a histology cassette at 60 °C. Cooled embedded tissues were cut into 6 µm sections and placed on ITO (indium tin oxide)-coated glass slides for MALDI imaging mass spectroscopy (Bruker Daltonik, Germany).

### Sample preparation and peptide substrate solution

Tissue sections were dried at room temperature or incubated and dried under atmospheric conditions for 60 min at 60 °C for heat inactivation (or partial attenuation) control experiments. Briefly, sections were dewaxed by passing them through decreasing concentrations of ethanol according to a protocol adapted from^20^.

For the peptide substrate solution, 19 custom made peptides (Tab. 1) with a purity of at least 90% (HPLC) were manufactured by JPT Peptide Technologies GmbH (Berlin, Germany) using standard solid-phase Fmoc chemistry. Peptide #20 was purchased from Merck KGaA (order number SCP0229, Merck KGaA, Darmstadt, Germany). 1 mg of each peptide was resuspended in molecular grade water and mixed together to obtain a concentration of 1 mM for each peptide in the resulting solution. For spray application, the substrate mix was diluted to a working concentration of 10 µM in 20 mM ammonium bicarbonate/acetonitrile 9:1).

**Table 1:** Applied substrates and expected enzyme conversion products.

Substrate peptide		Mass	Int.	Known target enzymes	Source (UniProt identifiers)	Product peptide 1	Product mass 1	Int.	Product peptide 2	Product mass 2	Int.
**Proteases**											
1	GTPGPQGLLGAPGI	1,234.42	−	MMP	CA21_HUMAN (P08123); CA21_MOUSE (Q01149)	GTPGPQG	612.64	−	LLGAPGI	639.80	−
2	GDQGPPGIPGQPGF	1,323.44	−	MMP	CA14_HUMAN (P02462); CA54_HUMAN (P29400)	GDQGPPG	626.62	−	IPGQPGF	714.82	−
3	GLAQPVGINTSTTC	1,361.52	+	MMP	SY07_HUMAN (P80098)	GLAQPVG	640.74	−	INTSTTC	738.81	−
4	GQGPGPKRGTEPKV	1,407.58	+++	Kallikrein-related peptidases	PCO1_HUMAN (Q15113)	GQGPGPK	639.71	+	RGTEPKV	785.87	+
5	KSYELPDGQVITIG	1,519.71	−	Caspase	IBP3_HUMAN (P17936); IBP3_PIG (P16611)	KSYELPD	850.92	−	GQVITIG	686.79	−
6	VSRLRAYLLPAPPA	1,523.87	++	MMP 2	ACTA_HUMAN (P62736); ACTA_MOUSE (P62737)	VSRLRAY	864.02	+	LLAAPPA	677.85	+
7	AIQTVADGLKKQEE	1,529.69	+	Caspase	KRAC HUMAN (P31749)	AIQTVAD	716.79	−	GLKKQEE	830.90	+
8	PGFSPFRSSRIGEI	1,549.77	++	Kallikrein-related peptidases	KNG_HUMAN (P01042)	PGFSPFR	806.92	+	SSRIGEI	760.85	+
9	MGRGHARLVHVEEP	1,587.81	++	MMP	A2MG_HUMAN (P01023)	MGRGHAR	783.91	−	LVHVEEP	821.90	−
10	EQVADIDGQYAMTR	1,596.73	++	Caspase	CTNB_HUMAN (P35222); CTNB_MOUSE (Q02248)	EQVADID	788.81	−	GQYAMTR	825.92	−
11	LEERPAVMTSPLYL	1,618.91	+++	MMP 2	FGR1_HUMAN (P11362); FGR1_MOUSE (P16092)	LEERPAV	812.92	−	MTSPLYL	823.99	−
12	QFWSLAAPQRFGKK	1,663.95	++	Tissue plasminogen activator, t-PA	NPFF_HUMAN (O15130); NPFF_MOUSE (Q9WVA8)	QFWSLAA	821.93	+	PQRFGKK	860.02	+
13	RRPKPQQFFGLMGK	1,690.05	++	MMP 9	TKN1_HUMAN (P20366); TKN1_MOUSE (P41539)	RRPKPQQ	909.06	−	FFGLMGK	798.99	−
14	YEVHHQKLVFFAED	1,761.95	+	MMP	A4_HUMAN (P05067); A4_RABIT (Q28748)	YEVHHQK	940.03	−	LVFFAED	839.92	−
**Kinases**											
15	RIRTQS*FSLQE	1,364.53	++	Kinases AKT1, CAMK2	Nitric oxide synthase, endothelial	RIRTQSpFSLQE				1,444.43	+
16	ELQDDY*EDLLE	1,381.41	−	Kinases SYK, LYN	Band 3 anion transport protein	ELQDDYpEDLLE				1,461.31	−
17	NKRRGS*VPILR	1,295.56	+++	Protein kinase A	Erythrocyte membrane protein band 4.2	NKRRGSpVPILR				1,375.46	−
18	LLRGPS*WDPFR	1,343.57	++	Kinases MAPKAPK-2, PKG1	Heat shock 27 kDa protein	LLRGPSpWDPFR				1,423.47	+
**Phosphatases**											
19	DADEYpLIPQQG	1,327.00	+	PTP1B, TC-PTP, SHP-2, Tyrosine	Epidermas growth factor receptor	DADEY*LIPQQG				1,248.31	
20	MCA-GDAEYpAAK(DNP)R	1,530.55	+	Protein Tyrosine Phosphatase Sigma-Aldrich: SCP0229		MCA-GDAEY*AAK(DNP)R				1,451.55	+

p, phosphoryl group; *, phosphorylation site; MMP, matrix metalloproteinase; Int., mass signal intensity: − no, + low, ++ intermediate, +++ high.

An automated spraying device (ImagePrep, Bruker Daltonik, nine spraying cycles) was used to apply 200 µl of this substrate peptide mix solution onto the sections over 20 min at room temperature. After tissue incubation at 37 °C for 5, 15, 60, or 120 min in a moist chamber, the matrix solution (1 ml 7 g/l HCCA in 50% ACN and 1% TFA) was applied using the ImagePrep device (15% power, ± 40% modulation, 60 spraying cycles). For the negative control, 200 µl 20 mM ammonium bicarbonate/acetonitrile 9:1 (without peptide mix) was applied in the same way, followed by matrix application.

### MALDI imaging analysis

MALDI imaging data acquisition was performed with a mass detection range of m/z 400–2,200, 200 laser shots per spot, sampling rate of 1.25 GS/s, and raster width of 100 µm or 50 µm on a Rapiflex MALDI/TOF using flexControl 3.0 and flexImaging 3.0 (Bruker Daltonik). External calibration was performed using a peptide calibration standard (Bruker Daltonik). Spectra were processed using in flexAnalysis 3.0 (Bruker Daltonik).

Statistical data analysis was performed using SCiLS Lab software (Version2020a, SCiLS GmbH, Bremen, Germany). MALDI-IMS raw data were imported into the SCiLS Lab software and converted to the SCiLS Lab file format. Simultaneous preprocessing of all data sets was performed for better comparability of the sample sets. Imported data were preprocessed by convolution baseline removal (width: 20). Peak finding and alignment were performed using a standard pipeline with the following settings: ± 0.156 Da interval width, mean interval processing, and medium smoothing strength^21–23^.

After MALDI imaging experiments, matrix was removed with 70% ethanol and tissue sections were stained with a hematoxylin/eosin (HE) fast staining kit (Roth, Germany).

## Results

To demonstrate the feasibility of EA-MALDI-IMS, or EA-IMS for short (Fig. 1), we generated 20 natural, protein-derived peptides (> 90% purity by HPLC, see supplements), as shown in Table 1, that were selected from a commercial enzyme substrate library (JPT Peptide Technologies GmbH, Berlin, Germany). After preliminary experiments with three peptide substrates of eight amino acids in length, as used for the commercial enzyme substrate kit (data not shown), we realized that the bulk of MALDI background signal of non-digested tissue sections was below 600 Da. Thus, the peptide lengths were increased by extending their sequences at both ends, according to the parent protein sequences such that their masses and the masses of the resulting products would be above 600 Da.

These peptides were pooled in equal concentrations to generate a peptide library mix that was spray-applied to the tissue slides. For the enzyme conversion step, the tissues were incubated in a humidity chamber at 37 °C for different incubation times. Figure S1 shows examples of average peptide spectra (max intensity; skyline) obtained by MALDI-IMS analysis after 60 min of incubation for kidney tissue samples with applied substrates, with substrates but on heat-inactivated tissue, and for samples without substrate application. No obvious differences were observed between MALDI mass spectra with the substrates ranging from 1,234 to 1,761 Da for the active tissue slices in comparison to the heat-inactivated samples (1 h at 60 °C), but the signal peaks for individual peptides were different although all peptides were applied at the same concentrations. In contrast, the enzyme product peaks developed more strongly in the noninactivated tissue samples. As expected, the mass spectra of tissue samples without substrate addition had no enzyme substrate mass peaks, but contained background peaks (e.g., matrix adducts).

**Figure 2:**
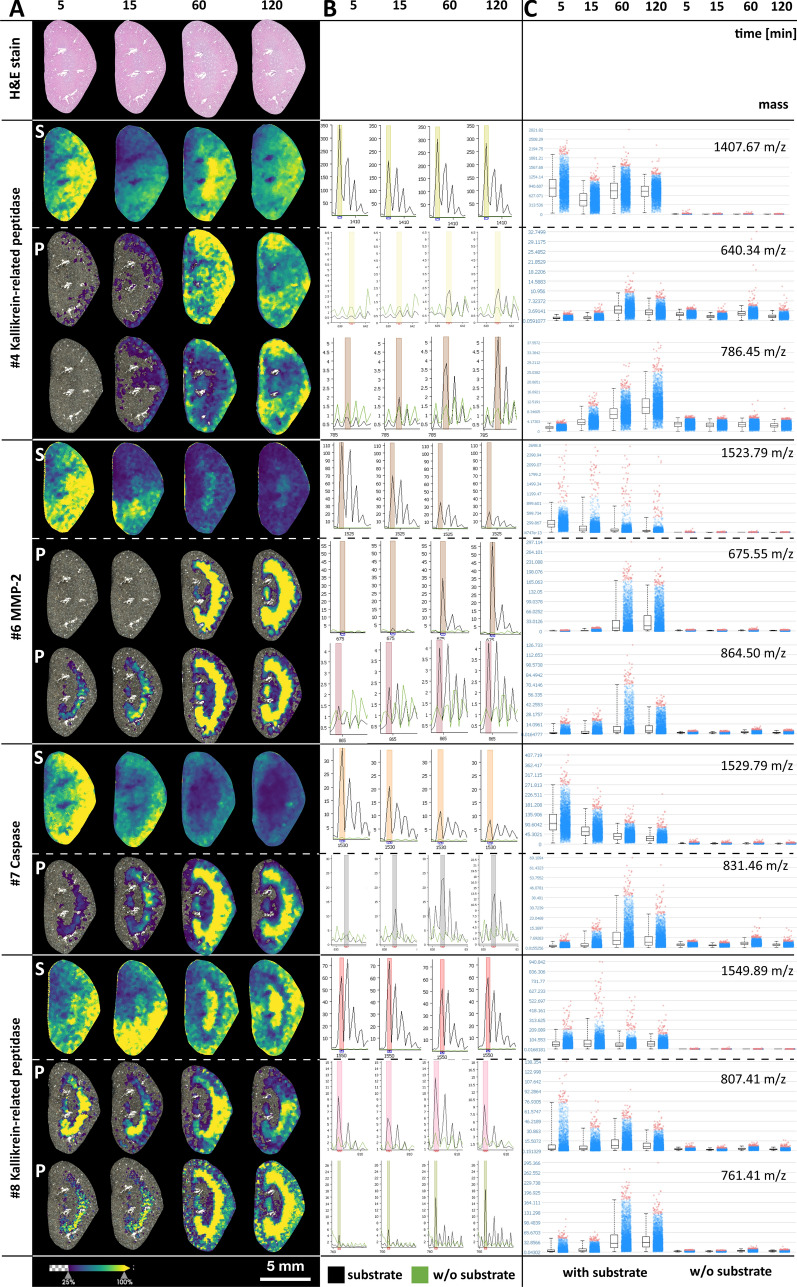

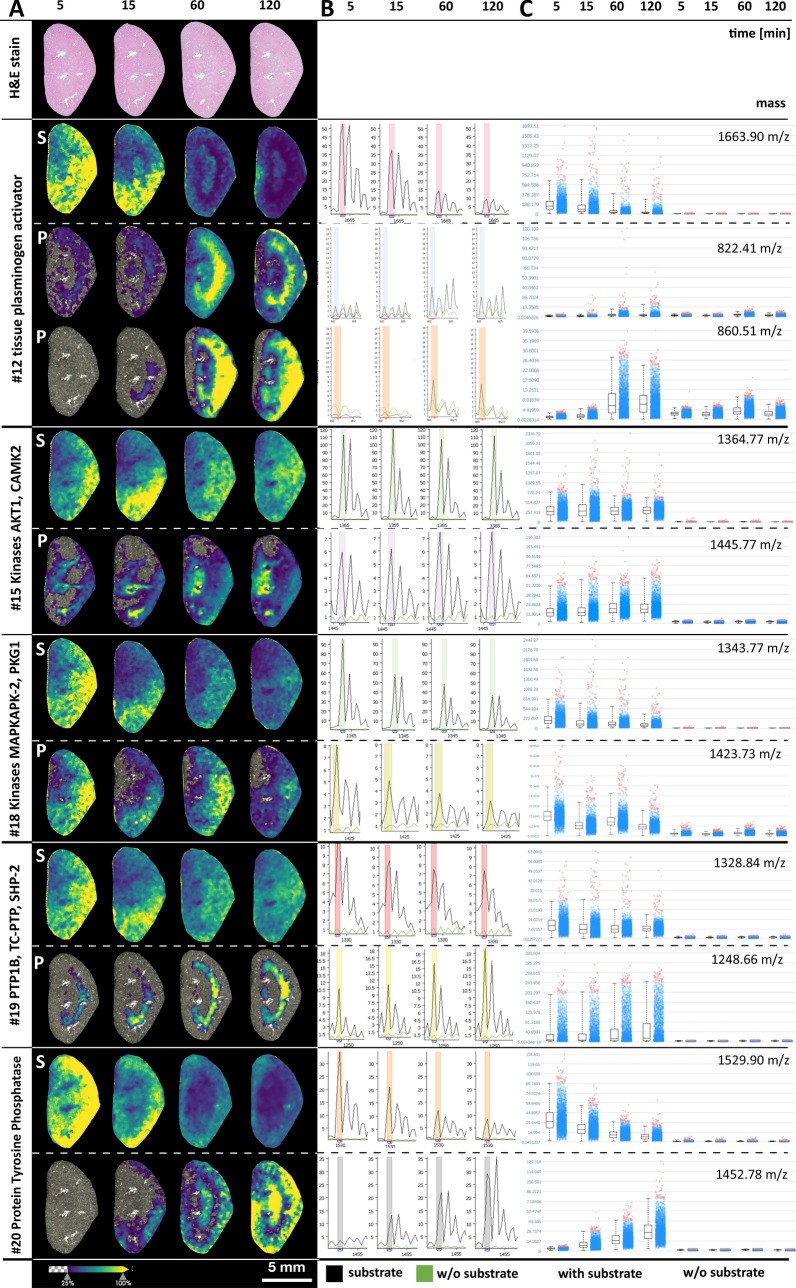
(1) and (2) Tissue maps, spectra, and dot plots of time-dependent enzyme product generation. (**A**) Heat maps of substrates and mass signal generation of detectable enzyme products after 5, 15, 60, and 120 min of incubation. (**B**) Corresponding mean mass spectra of the whole kidney areas with (black spectra) and without application of peptide substrate mix (green spectra). (**C**) Dot plots and corresponding box plots of each mass signal pixel with and without peptide mix.

**Figure 3:**
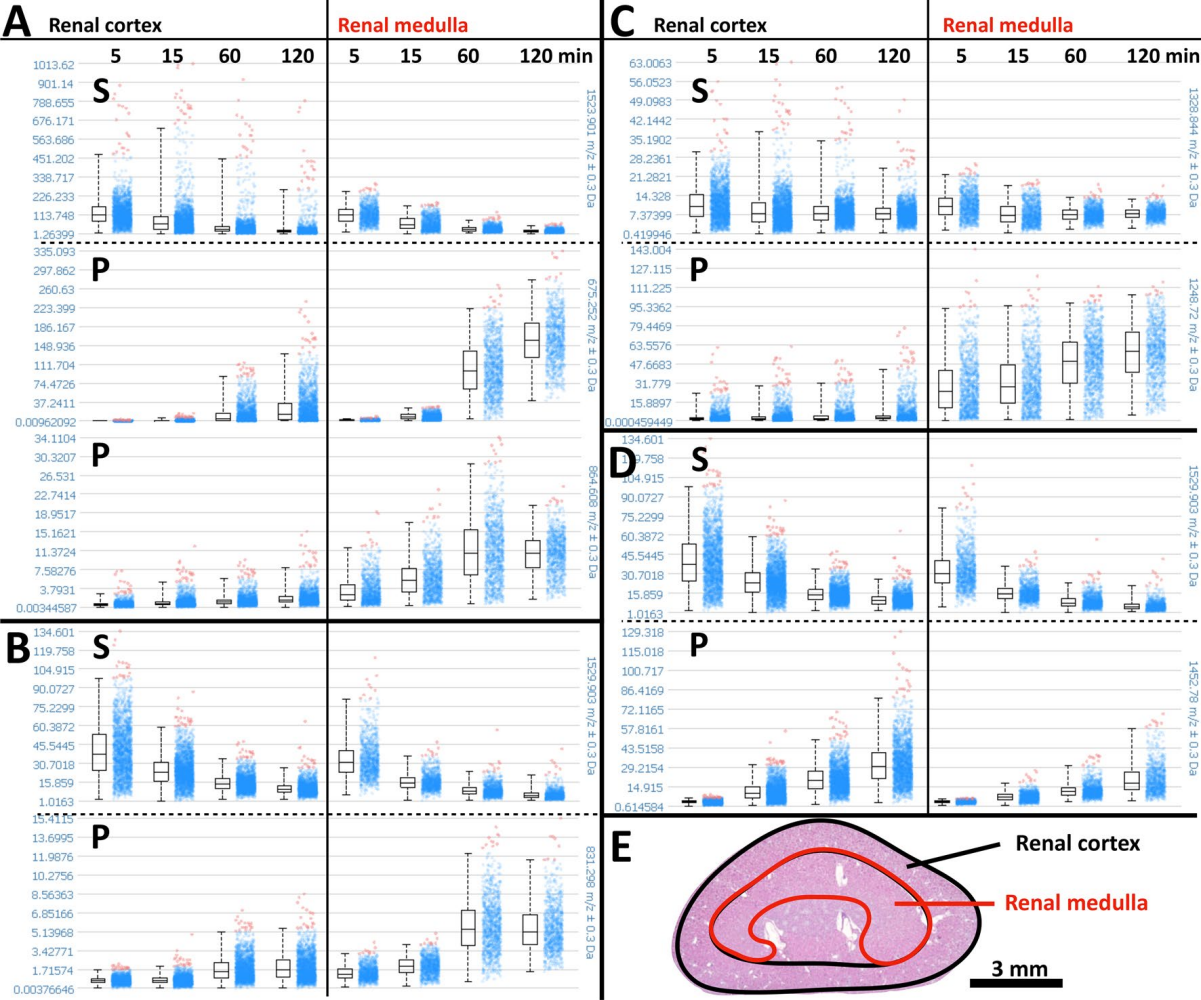
Comparison of enzyme substrate conversion in the renal cortex and medulla region of selected enzymes with regional differences. The spectral mass intensity dot plots and their corresponding box plots in the left columns represent the cortical region and those in the right columns the medulla as shown in (**E**). The first rows (S) represent the time-dependent intensities of the substrate masses and the second (and third) rows the corresponding product intensities (P). Enzyme substrate peptides are (**A**) #6 MMP-2, (**B**) #7 caspase, (**C**) #19 PTP1B, TC-PTP, SHP-2, and (**D**) #20 protein tyrosine phosphatase. (**E**) Representative H&E stain of a kidney section (after 5 min of incubation) with ROIs used for quantifications in (**A**) to (**D**).

Figure 2-1,2 present spatial peptide intensity maps of detectable enzyme substrate and product pairs including proteases, kinases, and phosphatases (see also Table 1). After spray application of the substrates, the kidney tissue specimens were incubated at 37 °C for 5, 15, 60, or 120 min, followed by matrix application and MALDI-IMS measurements. Most spatial intensity distributions of detectable product peptides demonstrate an increasing intensity up to 60 min of incubation time in comparison to controls without substrate mix. After an incubation time of 120 min, most product peptides showed a decrease or no substantial further increase. Product development varied for different enzyme substrates but was similar when two of the corresponding peptide products could be detected (Fig. 3A). Product peptide generation of one group of enzymes (MMP-2 #6, caspase #7, t-plasminogen activator #12, and the PTP1B phosphatase substrate #19) was strong in the renal medulla, whereas others (kallikrein-related peptidase #4, kallikrein-related peptidase #8, and the protein tyrosine phosphatase #20) were strong subcortically. Figure 2B shows the mean intensity MALDI mass spectra of the generated enzyme products integrated over the complete kidneys, and in Fig. 2C, peptide values of each product signal pixel are represented as dot plots with corresponding box plots and compared with the corresponding tissue sections without substrate mix.

For selected enzyme substrates with spatially different product generation between the renal cortex and medulla, substrate conversion to products in these two regions was analyzed individually and is shown in Fig. 3. The kinetics of enzyme product generation differed between regions and substrates and revealed the expected enzyme substrate conversion with time, including the two expected cleavage products of the protease. However, we also saw inconsistencies that hint at other factors.

**Figure 4:**
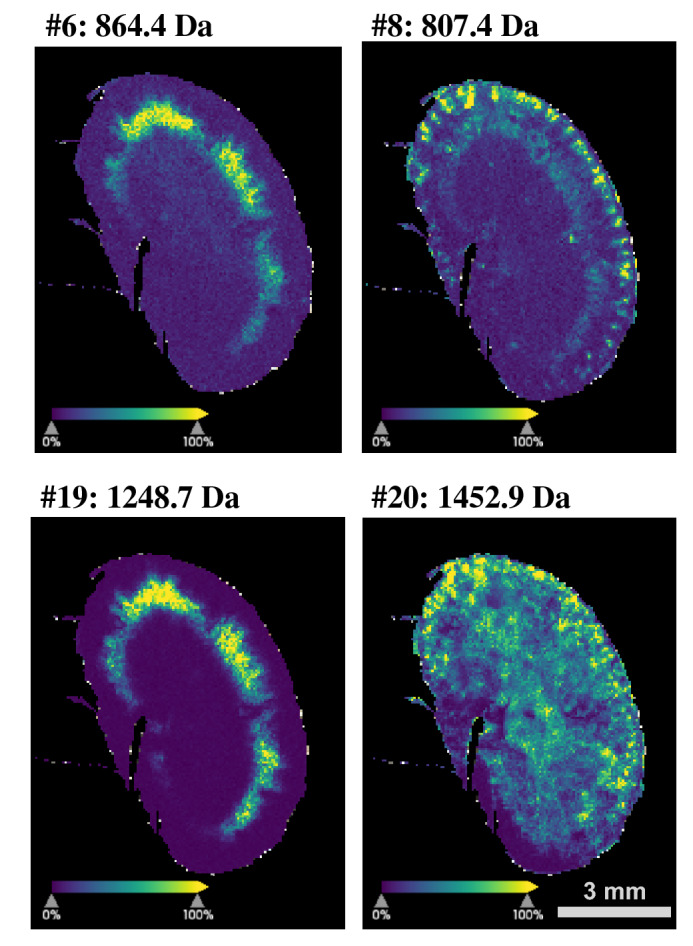
Enzyme conversion product maps at higher resolution. To demonstrate the potential of the method, four examples of enzyme conversion products are presented that were recorded at 50 µm resolution after 60 min of incubation. These maps reveal greater detail than the maps presented in Fig. 2 (100 µm resolution).

Spatial intensity distribution maps of detectable enzyme substrates and products in comparison to control experiments of corresponding tissue samples without substrate and with substrate application to heat-inactivated tissue samples are presented in the supplementary Figures S2 and S3. Overall, the results in these controls supported the observation that the detected substrate masses actually originated from the peptide substrate mixture, and that enzyme product generation was mostly sensitive to heat inactivation. Finally, we used the same setup for acquisition at the higher resolution of 50 µm (Fig. 4) after 60 min of incubation, which revealed a promising richness in detail that can be exploited in future correlative studies. The setup presented here resulted in an approx. four-fold increase in MALDI-IMS acquisition time at the higher resolution.

## Discussion

The purpose of this study was to demonstrate the concept of spatially measuring multiple enzyme activities by applying a substrate mix onto tissue sections with active enzymes and then measuring the generated enzyme conversion products together with the applied substrates using MALDI-IMS (Fig. 1). Criteria for substrate selection were that the targeted enzyme conversion would modify substrate masses (all enzymes except isomerases) and that the masses of the substrates and the expected products would differ. We chose an arbitrary set of 20 known enzyme substrates targeted to proteases, kinases and phosphatases from the literature^24,25^. These peptides were mixed in equal concentrations and spray-applied to paraffin-embedded sections of mice kidney that were zinc-fixed to preserve their enzyme activities based on the good results we and others achieved with fluorescence zymography of zinc-fixed tissue sections in the past^2,6^. Other possible fixation options include ethanol fixation and the use of frozen sections^2^. Tissue sections were incubated at 37 °C for 5, 15, 60, and 120 min to allow the enzyme catalysis to occur. A minimum incubation time of 5 min was assumed for processing the tissue slides. Moreover, depending on enzyme temperature characteristics, some substrate conversion may have occurred during spray application at room temperature.

We identified the expected masses of 16 of the 20 enzyme substrate peptides (see Table 1: column ‘Int.’ and Fig. S2), which supports the assumption that peptide detectability by MALDI-IMS strongly depends on the peptide properties determined by its sequence. The amino acid composition can influence all steps of MALDI-IMS by affecting tissue adsorption, MALDI desorption, and ionization characteristics, as is apparent from the differences in detection intensities of the substrates, which were all applied at the same concentration (Fig. S1). Asakawa et al.^26^ tested the influence of peptide compositions and demonstrated that, for instance, basic residues such as arginine and lysine, especially when located at the N-terminus, and phenylalanine enhanced the ion yields of peptides. In fact, unlike most of the detected peptides in our study, undetected substrate peptides #1, #2, and #16 had no arginins or lysins. Failure to detect peptides is likely to also occur in conventional trypsin-based MALDI-IMS—especially for low-abundance peptides. We applied 20 substrate peptides and detected the masses of 12 expected enzyme products. For most of the proteases, we detected both corresponding cleavage products with increasing intensities during the first hour of incubation. For caspase substrate #7 we identified only the protease product that contained lysines and missed the other, which lacked both lysine and arginine. Besides protease products, we detected masses of two of four kinases and both phosphatase products, underlining the potential of EA-IMS to investigate spatially related signaling pathways. Enzyme product generation increased up to 60 min of incubation, but was roughly stagnant or decreased at 120 min. This could be due to a decreasing product generation following substrate depletion or product inhibition along with unspecific product and substrate peptide degradation, e.g., by neprilysin^27–29^.

As it was the purpose of this preliminary study to demonstrate the concept of EA-IMS, it was beyond our scope to analyze or confirm the generation of individual enzyme products in greater detail. Nevertheless, enzyme expression of the detected enzyme products as observed in our study has been reported for the kidney before by investigators using other methods. Kallikrein-related peptidases or tissue kallikreins (KLKs) are a group of 15 known proteases that are expressed throughout the human body and play important roles in the tissue homeostasis and in disease^30,31^. While all known KLKs are expressed in the kidneys, the protein expression of KLK1, KLK3, KLK5, KLK6, KLK9, and KLK10 can be moderate to high. Remarkably, the KLK expression is lowered or lost in cancerous tissue^32^. Thus, investigation of renal cancer development could become a useful target for EA-IMS and specifically for imaging KLK activities. Interestingly, patterns of the detected KLK cleavage products of the substrate peptides #4 and #8 (Table 1 and Fig. 2) were different with cleavage products of substrate #4 occurring throughout the kidney with some preference for the cortex while those of substrate #8 occurring predominantly in the renal medulla and the subcapsular cortex. Among the several substrate peptides that were applied for MMPs, we detected both cleavage products in the intermediate region only for the MMP-2 substrate peptide #6. While MMP-9 expression is limited to the glomeruli^33^, MMP-2 is expressed throughout the nephron and more markedly in the tubuli^2,33^. Thus, the region of high MMP-2 product generation could correspond to an area of high tubular density. Additionally, we observed the two protease cleavage products of the tissue plasminogen activator (tPA). tPA is a serine protease that regulates blood fibrinolysis by converting plasminogen into biologically active plasmin and is involved in maintaining extracellular matrix homeostasis. Moreover, tPA acts as a survival factor that protects renal interstitial fibroblasts/myofibroblasts from apoptosis and is involved in the progression of kidney diseases^34,35^. We tested three caspase substrates but identified only one cleavage product of the caspase substrate peptide #7. Caspases are regulators and executors of apoptosis and thus, along with mitosis, maintain the normal tissue homeostasis, which explains basal levels of caspase activity in healthy kidneys^36^. Kinases and phosphatases are important elements in numerous signaling pathways. For example, MAPKAPK-2 (mitogen-activated protein kinase (MAPK)-activated protein kinase-2) and PKG1 (cGMP-dependent protein kinase 1), which are potential kinases for substrate #18, were identified as potential driver kinases involved in the development of clear cell renal cell carcinomas using peptide substrate microarray systems of fresh frozen tumor lysates without spatial information^37^. Furthermore, MAPKAPK-2 was shown to be involved in renal ischemia/reperfusion injuries^38,39^, and PKG1 has a wide range of physiological and pathological implications in the kidney^40^.

The different spatial distributions of enzyme product generation between kidney cortex and medulla we observed here and the prospect of further technical advances to enhance image resolution (Fig. 4) underline the potential of EA-IMS to spatially resolve and quantify multiple enzyme activities in the future, which in turn could contribute to the further elucidation of enzyme pathways and interactions. The exemplary mapping of kinase and phosphatase products in our study suggests that the built-in enzyme amplification has the potential to image activities of lowly expressed enzymes that could be missed with conventional non-amplifying MALDI-IMS.

### Limitations and future work

The purpose of this study was to demonstrate the concept of EA-IMS. We chose a set of 20 naturally derived peptide substrates with masses that were above the bulk of background mass signal of undigested tissue slices. One important aspect is that, unlike in conventional MALDI-IMS, which misses countless undetectable peptides as well, the peptides for EA-IMS (or other substrates in the future) are synthesized, which allows a broad range of modifications and optimizations. The design of the applied peptides could be freely modified to optimize their properties for imaging true enzyme activities while minimizing unspecific effects. Since many enzymatic reactions can be carried out by different enzymes at the same site, substrate specificity is a critical issue for interpretation of results and remains to be addressed. Besides adding arginines or lysins at the N-terminus^26^, artificial moieties could be added during synthesis, e.g., to reduce tissue adsorption and allow optimal desorption, to prevent unspecific proteolytic degradation, and to modify/increase the masses to optimal ranges without the need to elongate the peptide sequence, which in turn could increase the risk of unspecific degradation. Another possible target for improvement of the method is to optimize substrate concentrations of the substrate mix or of each substrate individually, which would not only modify the enzyme-to-substrate ratios and potential substrate inhibition but also influence the concentration of impurities from peptide synthesis (here up to 10%). Furthermore, the different tissue fixation methods and conditions could be tested and optimized, e.g., by use of frozen sections (although challenging due to difficult reproducibility in large-scale-studies and sample aging) or ethanol fixation. All of these possibilities for improvement could have substantial impact in terms of enhancing the sensitivity and robustness of EA-IMS and maximizing the number of enzymes that can be imaged in parallel.

## Conclusions

Our results demonstrate the principle feasibility of multiplex EA-IMS. Without optimization, peptide detectability was highly variable and some of the applied enzyme substrates and many of their products could not be identified. However, with the option of tailored design of peptide substrates including artificial moieties in conjunction with built-in enzyme amplification, it should be feasible to optimize the method and especially the substrates. This could ultimately lead to a tool for comprehensive in situ enzyme activity imaging or even spatially resolved quantification of enzyme activity.

## Supplementary information

Supplementary Information.
