# Supplementary material for: Multiplex enzyme activity imaging by MALDI-IMS of substrate library conversions
Source: Sci Rep. 2020 Sep 23;10:15522. doi: 10.1038/s41598-020-72436-2 (PMC7511933; doi:10.1038/s41598-020-72436-2)
Supplement: Supplementary file 1 — Supplementary Information. [file 41598_2020_72436_MOESM1_ESM.pdf]

Multiplex Enzyme Activity Imaging by MALDI-IMS of Substrate Library Conversions

Oliver Klein[2,4], Akvile Haeckel[1,4], Ulf Reimers[3], Grit Nebrich[2], Eyk Schellenberger[1*]

[1] Department of Radiology, Charité – Universitätsmedizin Berlin, Germany
[2] Berlin-Brandenburg Center for Regenerative Therapies, Charité – Universitätsmedizin Berlin, Augustenburger Platz 1, 13353, Berlin, Germany
[3] JPT Peptide Technologies GmbH
[4] These authors have equally contributed

*Corresponding author
Prof. Eyk Schellenberger
Department of Radiology
Charité – Universitätsmedizin Berlin
Charitéplatz 1
10117 Berlin, Germany
Phone +49 30 450 539013
eyk.schellenberger@charite.de

Supplementary material

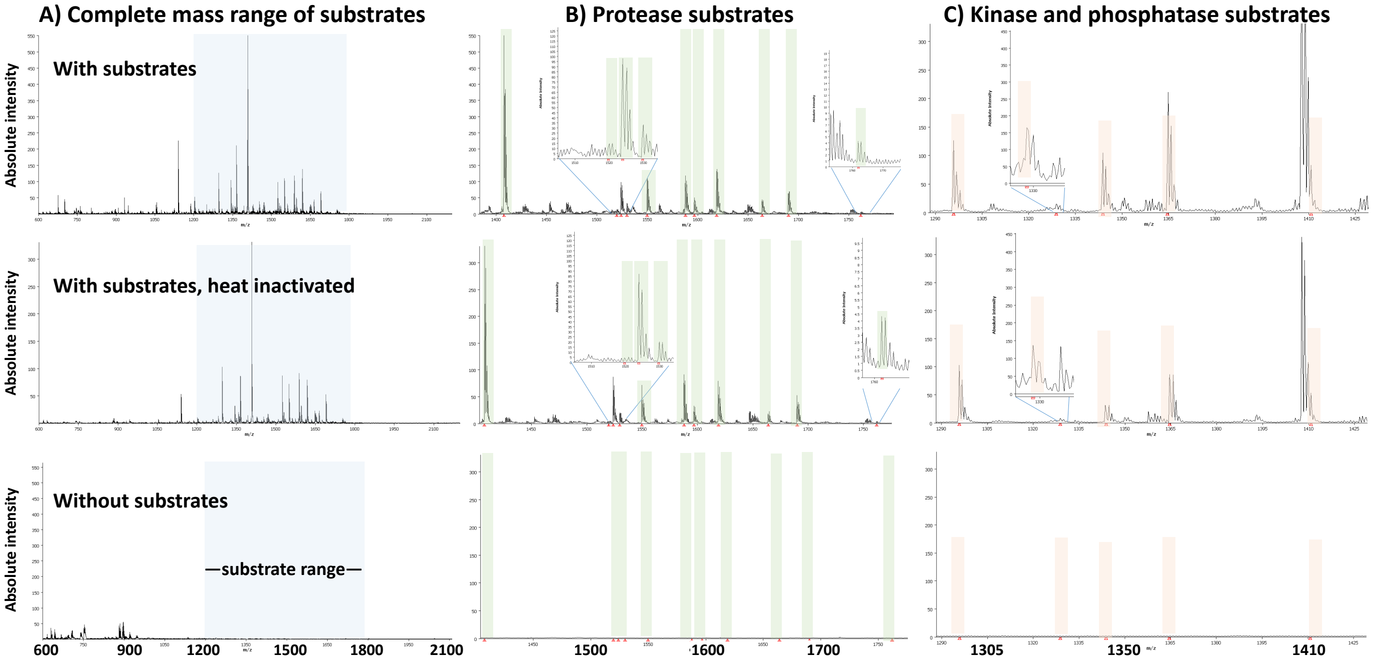

Figure S1: Examples of MALDI-IMS mass spectra of the substrate mix in comparison to background. (A) Complete mass range of all substrates; (B) Mass range of the protease substrates; and (C) mass range of the kinase and phosphatase substrates. The first row presents spectra of active tissue sections with the applied substrate mix after 60 min of incubation. Spectra in the second row are from heat-inactivated tissue sections with applied substrate mix. Spectra in the last row represent the low background mass signals without substrate mix.

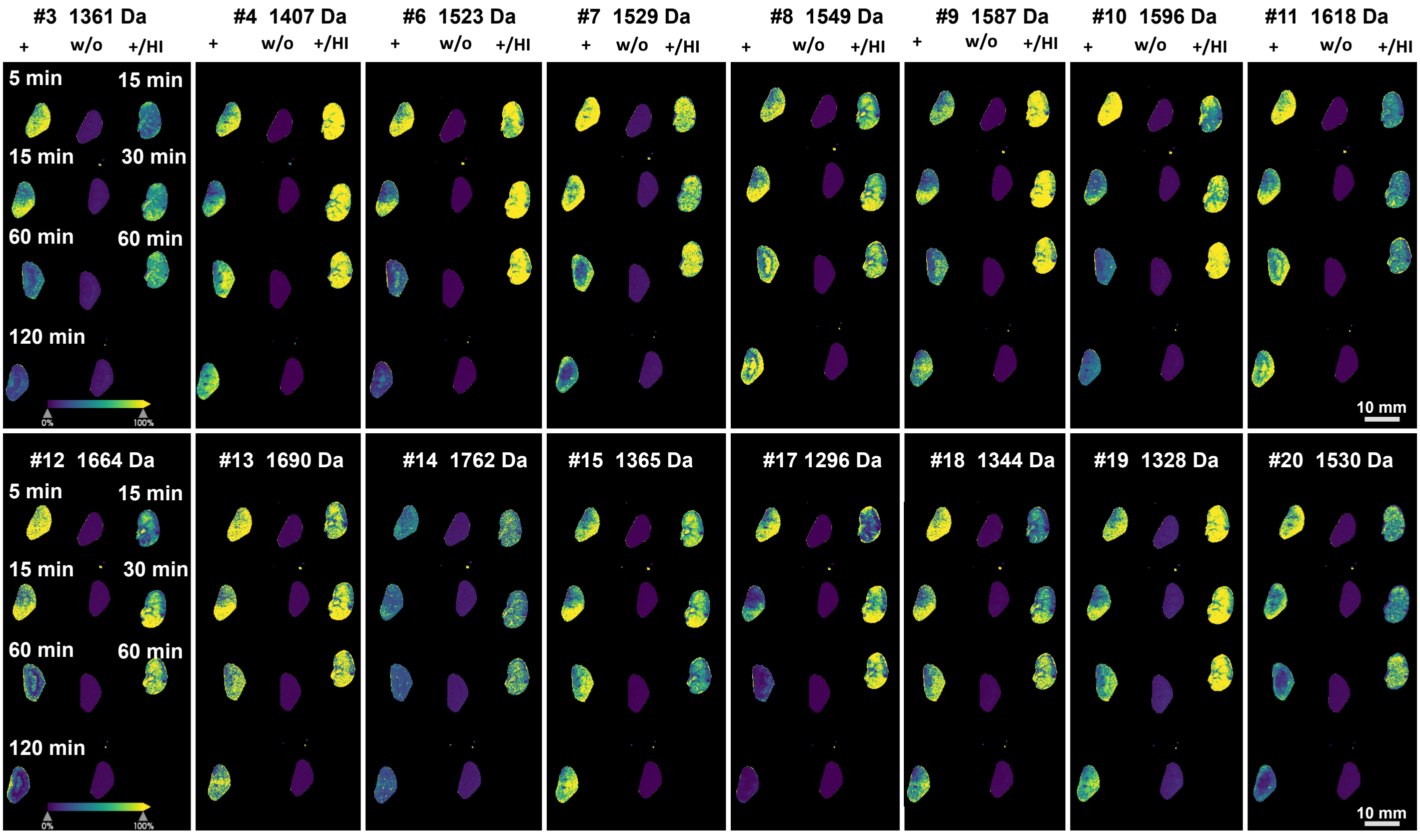

Figure S2: Maps of substrate masses detected in kidney sections and controls. Substrate mass maps with applied substrate mix **(+)**, without substrate mix **(w/o)**, and with substrate mix applied onto heat-inactivated tissue sections **(+/HI)**. Without substrate mix application, mass background signals are low.

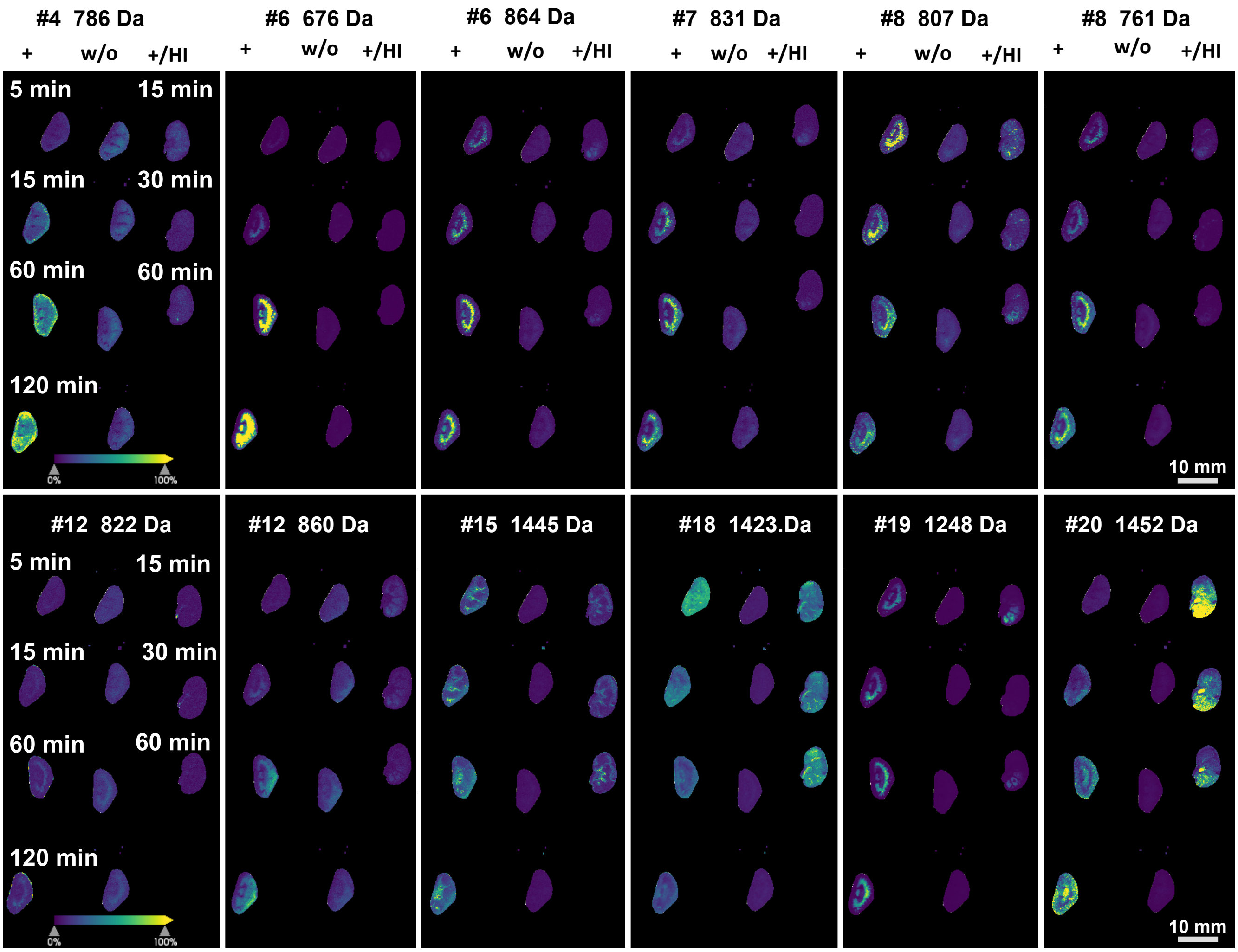

Figure S3: Maps of detected product masses detected in kidney sections and controls. Substrate maps with applied substrate mix **(+)**, without substrate mix **(w/o)**, and with substrate mix applied onto heat-inactivated tissue sections **(+/HI)**. For most substrates, no substantial products were generated on heat-inactivated tissue (drying for 1 h at 60°C), except for substrates #15, #18, and #20, where some product generation appeared to be preserved.
